# A Guide for Understanding and Designing Mendelian Randomization Studies in the Musculoskeletal Field

**DOI:** 10.1002/jbm4.10675

**Published:** 2022-09-20

**Authors:** April E Hartley, Grace M Power, Eleanor Sanderson, George Davey Smith

**Affiliations:** ^1^ MRC‐Integrative Epidemiology Unit Population Health Sciences, Bristol Medical School Bristol UK

**Keywords:** CAUSAL INFERENCE, EPIDEMIOLOGY, GENETIC EPIDEMIOLOGY, MENDELIAN RANDOMIZATION, STATISTICAL METHODS

## Abstract

Mendelian randomization (MR) is an increasingly popular component of an epidemiologist's toolkit, used to provide evidence of a causal effect of one trait (an exposure, eg, body mass index [BMI]) on an outcome trait or disease (eg, osteoarthritis). Identifying these effects is important for understanding disease etiology and potentially identifying targets for therapeutic intervention. MR uses genetic variants as instrumental variables for the exposure, which should not be influenced by the outcome or confounding variables, overcoming key limitations of traditional epidemiological analyses. For MR to generate a valid estimate of effect, key assumptions must be met. In recent years, there has been a rapid rise in MR methods that aim to test, or are robust to violations of, these assumptions. In this review, we provide an overview of MR for a non‐expert audience, including an explanation of these key assumptions and how they are often tested, to aid a better reading and understanding of the MR literature. We highlight some of these new methods and how they can be useful for specific methodological challenges in the musculoskeletal field, including for conditions or traits that share underlying biological pathways, such as bone and joint disease. © 2022 The Authors. *JBMR Plus* published by Wiley Periodicals LLC on behalf of American Society for Bone and Mineral Research.

## Introduction

Identifying causal risk factors for common conditions in the musculoskeletal field is key to developing interventions aimed at treating or managing these. Two frequent types of bias limit causal inference using conventional epidemiological methods. These are confounding^(^ ^1^ ^)^ and reverse causation.^(^ ^2^ ^)^ For example, in a study identifying a positive relationship between bone mineral density (BMD) and osteoarthritis (OA) by logistic regression, the association could be explained by another factor confounding this relationship, such as body mass index (BMI), which can cause both higher BMD and OA.^(^ ^1^, ^2^, ^3^, ^4^ ^)^ Although we are able to adjust for known and measured confounders in a multivariable regression analysis, it is possible that there are unknown and/or unmeasured confounders, or measurement error of known confounders, still biasing this relationship.^(^ ^5^ ^)^ We are therefore unable to conclude that there is an effect of BMD on risk of OA. Similarly, if we were interested in the effect of physical activity on OA risk and observed a relationship using traditional regression‐based methods, we could not be sure whether the relationship reflects an effect of physical activity on OA risk or whether physical activity is influenced by the symptoms of OA (ie, the disease process).

Randomized controlled trials (RCTs) are considered the gold‐standard level of evidence in causal inference. However, RCTs are expensive and time‐consuming and therefore often short in duration. Hence, it is not always possible to alter an exposure sufficiently to affect the outcome. For example, in a trial to determine if lower BMI reduces OA risk, short‐term changes in BMI are likely to be minimal and unlikely to alter disease risk. Ethical considerations can also inhibit RCT implementation; for example, it would be unethical to perform a clinical trial to determine if take‐up of smoking or alcohol drinking impact risk of rheumatoid arthritis. **Mendelian randomization** (MR; Box 1) uses the properties of germline genetic variants, which proxy an exposure, to overcome these two limitations present in standard epidemiological analyses and strengthen causal inference.^(^ ^6^, ^7^, ^8^, ^9^ ^)^ As genetic variants are randomly assigned at conception, they should be independent of confounding factors and should not be influenced by the outcome later in life.^(^ ^6^, ^7^, ^8^, ^10^ ^)^ MR is often implemented using **instrumental variable** (IV; Box 2) methods, where the genetic variant proxying the exposure is the instrument. Therefore, MR is analogous to an RCT, with individuals randomized to a particular genotype, rather than an intervention. An obvious difference between an RCT and MR is the length of time of the intervention, with genetic variants exerting their effect across the lifecourse, whereas an intervention in a trial, as discussed above, is often short term. Therefore, canalization, which is the weakening of an adverse effect on an outcome due to developmental compensation, needs to be considered when interpreting results of an MR study.^(^ ^8^ ^)^

Box 1 **Key Terminology (highlighted in bold throughout the text)**

**Mendelian randomization (MR):** A genetic instrumental variable approach aiming to overcome the limitations of confounding and reverse causality present in standard epidemiological analyses.

**Instrumental variable (IV):** A variable strongly related to the exposure of interest but only related to the outcome via its association with the exposure.

**Heritable:** A trait where variation is at least partially explained by inherited genetic variants.

**Genotype:** The alleles an individual possesses at a particular locus, for example, SNP genotype = AA or GA.

**Single‐nucleotide polymorphism (SNP):** A change in nucleotide at a single position, which is found in at least 1% of a population.

**Alleles:** The different variants present at a particular genetic locus.

**Effect allele:** The allele for which the effect size (beta) refers to.

**Genomewide association study (GWAS):** A hypothesis‐free scan of all SNPs across the genome (normally several million) to determine if the frequency of one of the alleles is more common in those with disease compared with those without (or vice versa) or to determine if an allele is related to a continuous outcome.

**Linkage disequilibrium (LD):** Correlation between genotypes at two SNPs due to lack of recombination due to their close proximity.

**Assortative mating:** When individuals tend to pick partners who are more similar in terms of more than one socioeconomic or anthropometric trait (eg, education and height). This can result in spurious associations between effect alleles of variants related to these traits. This could lead to spurious causal effect estimates for, eg, educational attainment on osteoarthritis, through the correlation of educational attainment‐increasing alleles with height‐increasing alleles.

**Population stratification:** When disease and allele frequencies both vary between populations of different ancestries, leading to the disease and allele appearing related in a combined population.

**Dynastic effects:** Where a genetically predicted parental phenotype influences offspring phenotype, eg, parents with a genotype associated with higher educational attainment will be more highly educated and may be more supportive of their children's education. Therefore, parental genotype is a common cause of both offspring genotype through inheritance and offspring outcome via parent's education rather than the offspring's education.

**Horizontal pleiotropy:** When a genetic variant affects two different traits (eg, exposure and outcome) via two separate pathways.

**Vertical pleiotropy:** When a genetic variant affects one trait (eg, the outcome) via a biological pathway involving another trait (eg, the exposure). Can also be referred to as mediated pleiotropy.

**Polygenic or genetic risk score (PRS/GRS):** A continuous measure representing the summed number of disease‐ or trait‐associated alleles an individual has. Can be weighted by the effect of each SNP on the trait/disease in question.

**Winner's curse bias:** An SNP has an overinflated beta estimate due to chance, which automatically leads to a lower *p* value, and variants that are selected by *p* value threshold in a GWAS will overrepresent those where such sample variation push the *p* value downward.

**Palindromic SNP:** An SNP where the two alleles represent the complementary base pairs of DNA (ie, A and T or C and G). It can therefore be difficult to determine if the effect allele differs between data sets or whether the DNA has been sequenced from the alternate strand and therefore the complement has been sequenced in that population.

**Cis SNP:** An SNP located within a certain distance from a gene (usually 1 million nucleotides from the start or end of the gene).

**Trans SNP:** An SNP located outside the region required to be defined as a cis SNP.

Box 2 **Instrumental Variables**

MR is often performed using instrumental variable (IV) methods developed for econometrics. An IV is a variable robustly associated with the exposure in an analysis but with no direct effect on the outcome, except via the exposure, and which will not be influenced by confounders of the exposure‐outcome relationship. For example, in an RCT, randomization to treatment or placebo is the IV, as only those randomized to receive treatment will receive the treatment. Randomization will therefore strongly predict who is treated and will only influence the outcome (eg, disease prognosis) through taking the treatment. If randomization has been performed correctly, confounders will be equally distributed across the treated and control groups. In an MR, a genetic variant robustly associated with the exposure should not be influenced by confounders or the outcome as genetic variants are allocated independently of environment and other genetic factors (Mendel's laws of segregation and independent assortment). The figure below highlights the analogy between an RCT and MR with the example of determining if vitamin D affects bone mineral density (BMD).
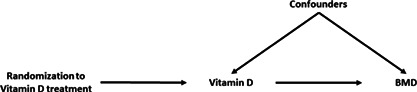
 
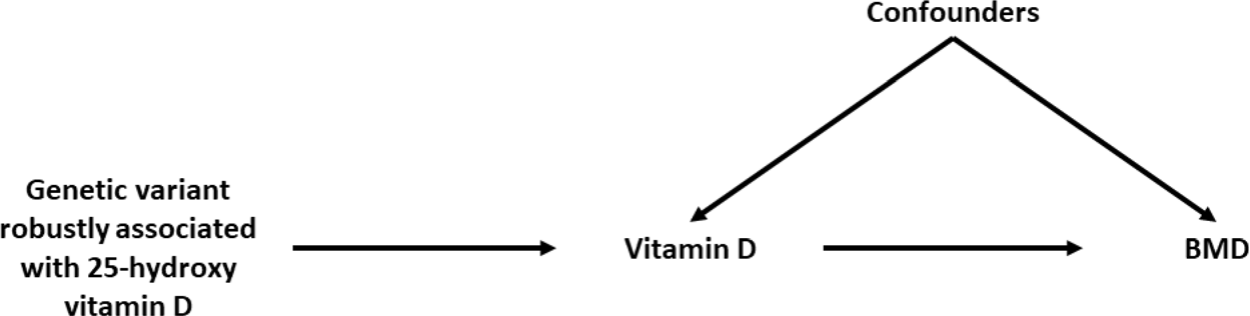

MR has been widely used in musculoskeletal research to identify causal risk factors for bone and joint disease. These uses have been extensively reviewed elsewhere.^(^ ^11^, ^12^, ^13^ ^)^ In this article, we aim to provide a guide to reading and interpreting MR studies in the musculoskeletal field, highlighting important methodological considerations and the main results that should be presented in an MR study. We then go on to review key considerations for musculoskeletal traits, including conditions or traits that share underlying biological pathways, such as BMD and OA. We hope this review makes MR studies more accessible to non‐geneticists who have an interest in determining causal risk factors for musculoskeletal conditions.

## MR: The Basics

### Instruments

MR uses genetic variants as IVs and is therefore applicable to any exposure that is **heritable**. IVs in an MR analysis are typically **single‐nucleotide polymorphisms** (SNPs), which are variations of a single nucleotide that occur commonly on every chromosome and at a frequency of greater than 1% in a population. The variable will be the genotype of the SNP, which is the number of copies (0, 1, or 2) of a particular **allele** (often known as the **effect allele**) an individual has. SNPs are normally selected as instruments when they are robustly associated with the exposure of interest, as identified by **genome wide association studies** (GWAS). A GWAS includes millions of statistical tests across the genome, and therefore a stringent *p* value is required to account for multiple testing and reduce the risk of false positives. As such, genomewide significance is often defined as a *p* value ≤5 × 10^−8^. As alleles for SNPs located close together on a chromosome tend to be inherited together in a process called **linkage disequilibrium** (LD), there are often many correlated SNPs identified at a locus at genomewide significance. A process called LD‐based clumping is employed to identify the most likely causal SNP at a particular locus to ensure independence of instruments. LD‐based clumping essentially scans a chromosomal region to identify the SNP with the smallest *p* value out of all the SNPs in that locus that are in LD (based on a predefined correlation threshold, eg, *r* ^2^ ≤ 0.001).

### Assumptions

**Fig. 1:**
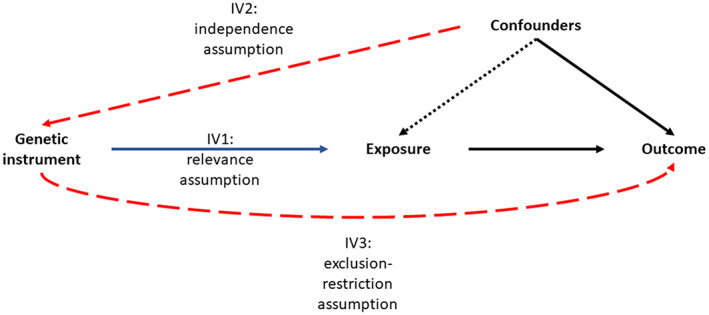
The three core assumptions of Mendelian randomization. Red dashed arrows represent pathways that should not exist if the instrument is valid. Blue arrows represent conditions that need to be true for an instrument to be considered valid. The black dotted arrow represents the fact that the confounder does not need to influence the exposure to violate instrumental variable 2 (IV2): the assumption is no confounding of the genetic instrument and the outcome.

**Fig. 2:**
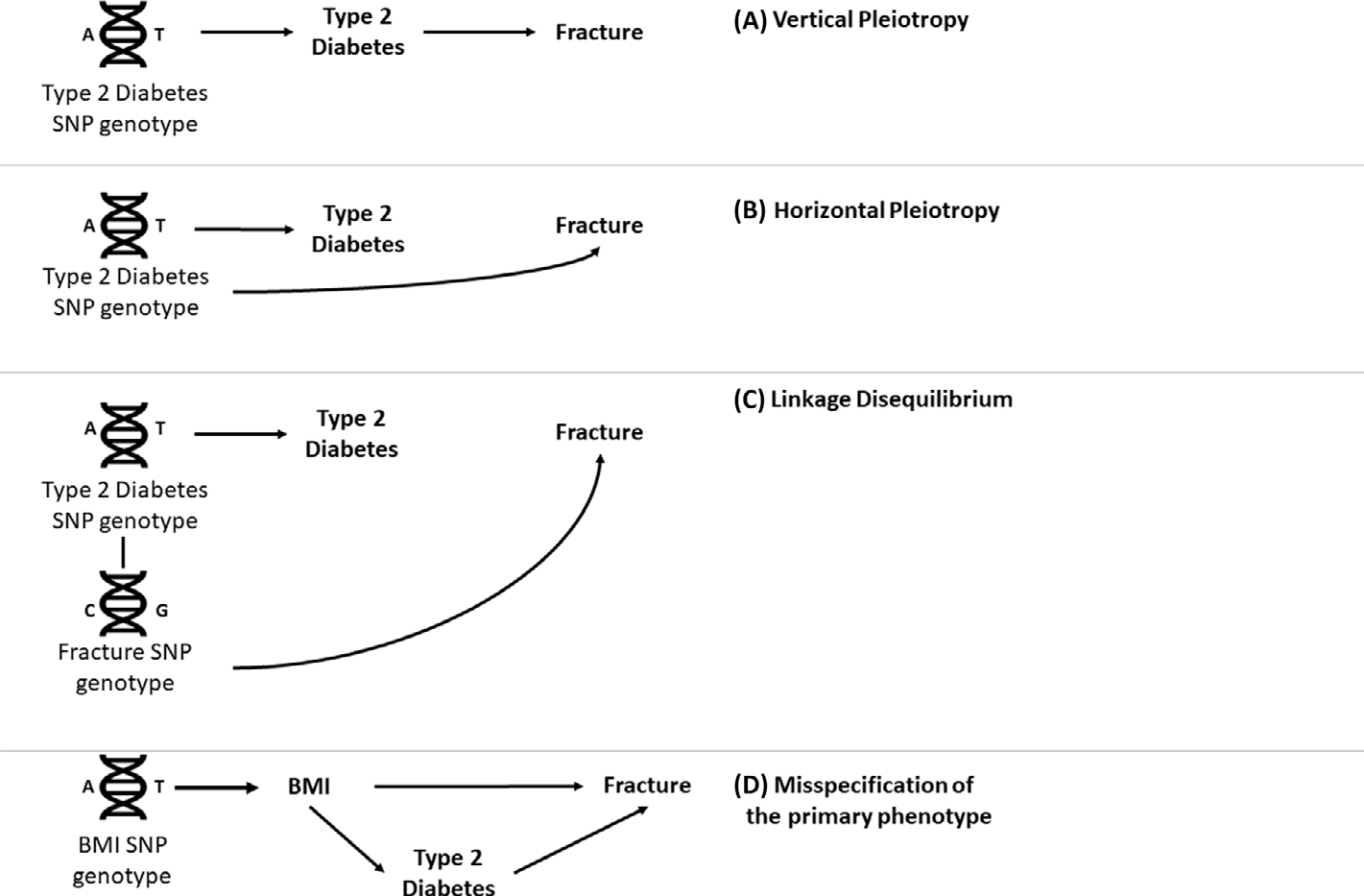
Directed acyclic graph highlighting vertical versus horizontal pleiotropy. (*A*) Vertical pleiotropy is where the instrument is only associated with the outcome (fracture) via the exposure (type 2 diabetes). This is a key requirement of Mendelian randomization (MR) and does not lead to biased effect estimates (except in the case of misspecifying the primary phenotype outlined below). (*B*) Horizontal pleiotropy is where the instrument is associated with the outcome via a pathway independent of the exposure. This will lead to bias in the effect estimate for type 2 diabetes on fracture. (*C*) Where the instrument is in close proximity to a single‐nucleotide polymorphism (SNP) strongly associated with the outcome, the genotypes at these two loci can be correlated due to linkage disequilibrium (LD). This will cause the same bias as horizontal pleiotropy in an MR study. It is important to note that although SNPs are often selected based on the lowest *p* value in a region, they are not necessarily causal for the exposure but are in LD with a disease‐causing variant. (*D*) Misspecification of the primary phenotype can cause bias in an MR analysis. This occurs when genetic variants associated with a phenotype upstream of the exposure are identified in the genomewide association study (GWAS) of the exposure due to a large sample size and thus well‐powered GWAS. For example, in an MR of type 2 diabetes on fracture, a positive effect of type 2 diabetes may be observed due to the inclusion of body mass index (BMI) SNPs as instruments for type 2 diabetes.

For MR to accurately determine if there is an effect of an exposure on an outcome, the IV(s) need to satisfy three core assumptions.^(^ ^7^, ^8^ ^)^ The three core assumptions, illustrated in Fig. 1, are:

- IV1: The instrument strongly predicts the exposure, also known as the relevance assumption. With multiple instruments, the assumption is that the instruments will collectively predict the exposure. This is normally satisfied by selecting instruments that are associated with the exposure at genomewide significance (as discussed in the Instruments section). It is important to note that the instrument does not necessarily need to cause the exposure; although the SNP with the lowest *p* value at a particular locus is usually selected as the instrument, this SNP could actually be in LD with the causal SNP.
- IV2: There is no confounding of the instrument‐outcome relationship, also referred to as the independence assumption. This can be violated due to **assortative mating, population stratification, or dynastic effects**.^(^ ^9^, ^14^ ^)^
- IV3: The instrument is not associated with the outcome independent of the exposure, also known as the exclusion‐restriction assumption. This can be violated when the instrument influences the outcome via a biological pathway not involving the exposure, which is known as **horizontal pleiotropy**. This should not be confused with **vertical pleiotropy**, whereby the genetic variant is associated with the outcome via a pathway involving the exposure (Fig. 2).

The majority of MR estimation methods assume a linear effect of the exposure on the outcome. An additional assumption is required to ensure accurate assessment of the magnitude of effect.^(^ ^7^ ^)^ One version of this assumption, known as the homogeneity assumption, assumes that the effect does not vary across strata of the population, for example, by age or sex,^(^ ^8^ ^)^ or that the effect of the exposure on the outcome does not change across levels of the instrument.^(^ ^9^ ^)^ If this assumption is met, the effect estimate can be interpreted as the average causal effect of the exposure on the outcome across the population.^(^ ^9^, ^15^ ^)^ An alternative, more relaxed and plausible version of this assumption is the monotonicity assumption, which assumes the direction of the effect of the instrument on the exposure is the same for everyone in the population, which means that the effect estimate can be interpreted as the average effect of differences in the exposure on the outcome for those whose exposure is associated with the instrument.^(^ ^15^ ^)^

Furthermore, for any MR study to estimate the effect that intervening on the exposure will have on the outcome, the assumption of gene–environment equivalence must hold.^(^ ^16^ ^)^ This means that the effect of a change in exposure due to a change in allele for the genetic instrument has the same effect on the outcome as an environmental or pharmaceutical intervention used to alter the exposure.^(^ ^16^ ^)^ This may be more likely to hold for directly measured traits such as biomarkers rather than complex social traits with a stronger environmental component, such as education status.^(^ ^9^ ^)^

### Individual‐level versus summary‐level approaches

MR analyses are commonly categorized into two forms in the literature: one‐sample (or single‐sample or individual‐level) and two‐sample (or summary‐level). Initial MR analyses, mainly published before 2014, used individual‐level data, where genotype, exposure, and outcome data were all available in the same population. However, this limited sample size and therefore statistical power, leading to the implementation of summary‐level approaches. Summary‐level approaches only require summary statistics from a GWAS of the exposure and outcome (Table 1), which are not performed on the same sample but under the assumption that the GWAS populations are drawn from the same underlying population. This allows researchers to use the largest available GWAS for each trait, maximizing sample size and thus statistical power. For example, there are few populations with both dual‐energy X‐ray absorptiometry (DXA) scans and radiographs for a sufficient sample size for an MR analysis of the effect of DXA‐assessed femoral neck BMD on OA. By using summary‐level MR, we are not limited to populations where both DXA scans and X‐rays have been performed and can utilize data available from large‐scale GWAS consortia. The statistical analysis performed differs between the two approaches (Table 1), although given sufficient sample size and strong independent instruments, the two methods should estimate the same effect.^(^ ^8^ ^)^

**Table 1:** Summary of the Two Approaches to Mendelian Randomization

	Individual‐level data MR	Summary‐level data MR
Requirements	Measured exposure, outcome in the same population. Genotype dosage for all instruments in the same population (or a polygenic risk score)	SNP‐exposure association results and SNP‐outcome association results from separate populations, including: Effect allele Other allele Effect allele frequency Beta for the per allele effect on the exposure or outcome (unit increase or log odds) Standard error for the beta
Possible analysis methods	To test for causal effect: linear/logistic regression of SNP genotype or polygenic risk score on the outcome To quantify causal effect: Single SNP: Wald ratio estimate β_outcome_/β_exposure_ Single/multiple SNPs/polygenic risk score: Two‐stage least‐squares regression	To test for causal effect: determine SNP‐outcome effect using summary statistics from a published GWAS To quantify causal effect: Single SNP: Wald ratio estimate Multiple SNPs: an inverse‐variance weighted meta‐analysis of the Wald ratio estimate for each SNP
Testing the relevance assumption	First‐stage *F*‐statistic	Mean *F*‐statistic for SNP‐exposure association
Testing the independence assumption	Associations between the instrument(s) and potential confounders can be directly tested for all known/measured confounders	N/A
Testing the exclusion‐restriction assumption	Sargan test for heterogeneity in individual SNP results	Cochran's *Q* statistic as a measure of heterogeneity in Wald ratio estimates MR‐Egger intercept as a measure of the average effect of the SNP on the outcome when there is no effect of the SNP on the exposure
Pleiotropy‐robust methods	MVMR MR‐GENIUS controls for some directional pleiotropy^(^ ^106^ ^)^ sisVIVE and adaptive LASSO for outlier removal^(^ ^107^, ^108^ ^)^	Several methods, including MR‐Egger, weighted median, weighted mode, MR‐CAUSE, MR‐PRESSO, MVMR, reviewed in Sanderson et al.(9) Can be broadly categorized as outlier adjustment, outlier removal, or estimate adjustment methods
Benefits	More flexibility in models (eg, can test for non‐linear effects) and covariates Ability to perform subgroup analyses (eg, sex‐stratified)	Larger sample sizes increase power Greater range of sensitivity analyses to determine pleiotropy‐robust estimates of causal effect
Limitations	Sample limited to those with measured exposure, outcome, and genotype, often restricting sample size Fewer methods to interrogate pleiotropy Weak instrument bias is toward the observational (confounded) estimate, potentially resulting in type 1 error	Assumes the two study populations are drawn from the same underlying population in terms of ethnicity, sex distribution, etc. Weak instrument bias toward the null, resulting in type 2 error Overlap in individuals between samples can result in bias toward the observational estimate in the presence of weak instruments (type 1 error) Unable to control which covariates are adjusted for Unable to perform subgroup analyses unless summary statistics are available for specific subgroups for both exposure and outcome

In the case where only one SNP has reached genomewide significance and is appropriate to instrument the exposure, presence of a causal effect can be determined by the effect of the SNP on the outcome, either by regressing SNP genotype on the outcome using individual‐level data or by extracting the summary statistics (beta, standard error and *p* value) from a published GWAS.^(^ ^17^ ^)^ This method determines if a causal effect is present but does not provide an estimate for the magnitude of this effect. The point estimate for the effect can be estimated by a Wald ratio, which is simply the beta for the SNP effect on the outcome divided by the beta for the SNP effect on the exposure.^(^ ^7^ ^)^ This can be calculated using both individual‐level and summary‐level data. Alternatively, for individual‐level data, it is also possible to estimate the effect using two‐stage least‐squares regression: the exposure is first regressed on the instrument, then the outcome is regressed on genetically predicted values of the exposure.^(^ ^18^ ^)^ The conventional standard error in the second model does not account for the additional uncertainty that arises from the exposure being predicted, rather than observed. Therefore, a correction needs to be applied to the standard error obtained from the second regression to correctly account for the uncertainty.^(^ ^9^ ^)^ This correction is performed as standard in MR and IV software packages (discussed in the section Software and data sources).

Given the availability of large‐scale biobanks such as UK Biobank and worldwide collaborative efforts, GWAS power has increased in recent years and often more than one SNP is an appropriate instrumental variable. In the individual‐level data setting, two‐stage least‐squares regression can still be used to estimate the effect with multiple SNPs, although it is often preferable to combine the SNPs into a **polygenic or genetic risk score** (PRS or GRS).^(^ ^19^ ^)^ This often increases the variance in the exposure explained and therefore the instrument strength. However, if any of the SNPs in the score violate either the IV2 or IV3 MR assumptions, described in Assumptions, the whole score will be an invalid instrument.

**Fig. 3:**
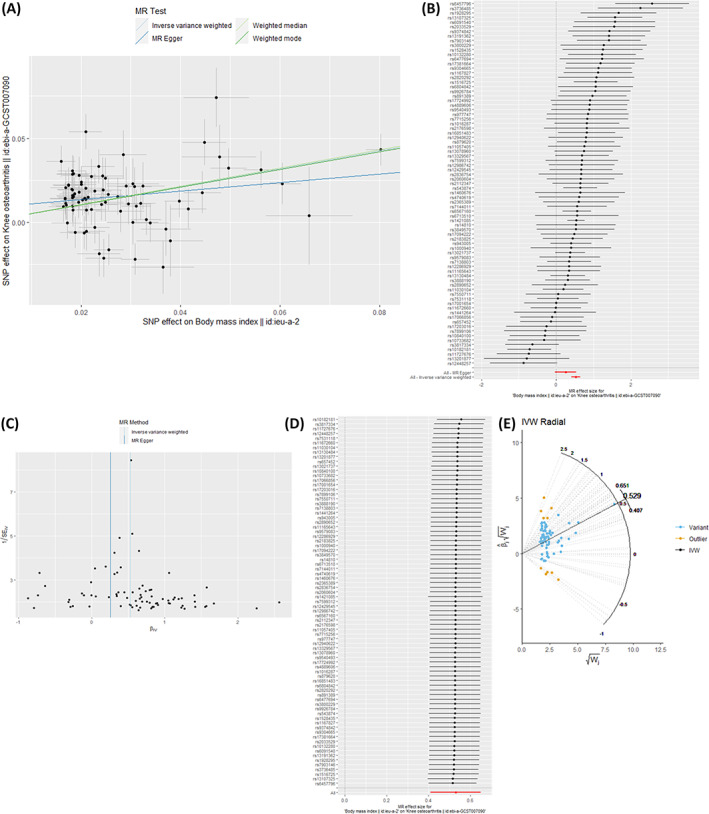
Results commonly presented in Mendelian randomization (MR) studies. (*A*) A scatterplot is often presented comparing results of the different summary‐level meta‐analyses. Here we are looking for consistency in the slope (ie, causal effect estimate) between the different approaches, which we do observe. The MR‐Egger slope is slightly weaker due to the intercept not being fixed at 0, although the intercept is close to 0 and the slope is similar in direction and magnitude to the other methods, meaning we can be more confident in the inverse‐variance weighted (IVW) causal effect estimate. (*B*) A single‐nucleotide polymorphism (SNP) analysis can be performed to detect heterogeneity, with each point on the forestplot representing the Wald ratio for an individual SNP. Here we do observe heterogeneity in effect estimates, which means we should be cautious of horizontal pleiotropy, but the majority of SNPs do provide causal effect estimates in the positive direction. (*C*) The funnel, or volcano, plot shows the Wald ratio for each SNP on the *x* axis and the weight on the *y* axis (ie, the inverse of the standard error). Asymmetry in this plot would indicate that an SNP has a large effect on the outcome, relative to its precision, indicating pleiotropy. As this plot is relatively symmetrical, we can be more confident in the IVW estimate. (*D*) A leave‐one‐out analysis can identify outlier SNPs with a large effect on the overall IVW estimate. If the effect estimate (indicated by the points, with horizontal bars indicating 95% confidence intervals) changes in magnitude and/or direction when an individual SNP is excluded, this is evidence that the SNP is an invalid instrument. In this case, there is no evidence to suggest any of the SNPs are outliers. (*E*) A radial plot of the SNP weight versus the beta times its weight can show outlier SNPs (in green), plotted alongside the IVW estimate. All plots have been generated using the TwoSampleMR^(^ ^42^ ^)^ and RadialMR^(^ ^109^ ^)^ packages and publicly available summary statistics^(^ ^110^, ^111^ ^)^ available through the IEU OpenGWAS project.^(^ ^43^ ^)^

For a summary‐level analysis, two‐stage least‐squares regression is not possible, and instead the effect is estimated by meta‐analyzing the individual‐SNP Wald ratios. The most common method used for this meta‐analysis is the inverse‐variance weighted (IVW) meta‐analysis, which can be implemented as a regression of the SNP‐outcome effect estimate on the SNP‐exposure effect estimate, weighted by the inverse of the standard error of the SNP‐outcome estimate^(^ ^20^ ^)^ (Fig. 3). Other methods are available for this meta‐analysis; these are commonly used as sensitivity analyses to test for horizontal pleiotropy and will be discussed in Sensitivity analyses relaxing the core assumptions.

An important consideration in a summary‐level MR analysis is trying to ensure that the two study populations are drawn from the same underlying population, or more specifically that the instrument‐exposure association is the same in the two samples, while reducing the overlap between the two samples. Overlap in samples can lead to correlation between SNP‐exposure and SNP‐outcome estimates, due to chance associations between the instrument and confounders of the exposure‐outcome relationship.^(^ ^21^ ^)^ This can result in bias of the causal effect estimate toward the observational estimate, which can lead to a false‐positive result.^(^ ^21^, ^22^ ^)^ Bias is greater when using weak instruments^(^ ^23^ ^)^ as the effect estimate from this GWAS may reflect **Winner's curse bias**.^(^ ^22^ ^)^ Similarly, in an individual‐level MR, if the instruments were identified in the same study population, there is a greater chance of Winner's curse and therefore bias toward the observational effect estimate. Methods such as block jackknife resampling, described in the section Novel Methods for Overlapping Samples, can overcome this problem.^(^ ^24^ ^)^

Weak instruments can also occur if the instruments are not genomewide significant in the data used for analysis. This could occur when using individual‐level data with instruments selected from a separate GWAS, or with summary‐level data if the GWAS used to obtain the SNP‐exposure associations is not the same as the one used to select the SNPs. Weak instrument bias will bias estimates toward the observational estimate in individual‐level MR or in summary‐level MR where the same samples are used for the exposure and the outcome. In a summary‐level MR analysis where the samples do not overlap, using weak instruments (eg, those selected from an unreplicated GWAS and therefore had a chance association with the exposure, or those associated with the exposure above genomewide significance) will lead to bias toward the null rather than the observational estimate as there will be no covariance between SNP‐exposure and SNP‐outcome estimates.^(^ ^25^ ^)^

Ancestry should be considered to ensure the two samples for a summary‐level analysis are drawn from the same underlying population. The instrument used may not be the causal variant and may have been identified in GWAS because it is in LD with the causal variant. Therefore, in populations of different ancestry where allele frequencies and LD structure differ,^(^ ^8^ ^)^ the SNP may not be associated with the exposure to the same extent in the exposure and outcome populations. Most large‐scale GWAS are currently performed on European samples, and there are fewer large collaborative efforts for non‐European populations.^(^ ^26^ ^)^ Although these large‐scale pan‐European collaborative efforts are useful to increase sample size and power for GWAS and reduce the risk of population stratification, generalizability of findings to other populations is limited.

### Pre‐analysis quality control

An essential step before summary‐level analysis is harmonizing the data set to ensure effect alleles match across the exposure and outcome GWAS. For example, the exposure data set may provide the beta for the effect of each additional copy of an A allele on the exposure, whereas the outcome data set may be based on the alternate C allele. This would lead to an incorrect direction of effect estimated by the Wald ratio for that SNP, overall biasing the IVW estimate. Where the effect alleles do not match between data sets, the alleles are flipped in one of the data sets by subtracting the beta from zero. This process is more difficult for **palindromic SNPs**: either A‐T as allele 1 and 2, respectively, or C‐G as allele 1 and 2. This is because it is unclear whether the alleles have been coded differently or whether the allele has been sequenced from the other DNA strand. The allele frequency of the coded allele can be useful here: if, for example, a C effect allele has an allele frequency of 0.3 in one data set and a G effect allele has frequency of 0.7 in the other data set, the two data sets used different effect alleles. Therefore, the beta needs to be converted in one data set so the effect alleles match. However, if the frequency of the C effect allele was the same as the G effect allele in the other data set, the two data sets have clearly used different DNA strands for sequencing and no change to the beta is required. If the allele frequency is close to 0.5, it is impossible to determine if the effect allele differs or if the reference DNA strand differs; therefore, these SNPs should be removed from analyses.

### Sensitivity analyses relaxing the core assumptions

Several methods are available and commonly used to test the core MR assumptions described above. Only IV1 can be explicitly tested, using the *F*‐statistic as a measure of instrument strength, which reflects the sample size and the variance in the exposure explained by the instrument (ie, the *r* ^2^).^(^ ^27^, ^28^ ^)^ A higher *F*‐statistic indicates a stronger instrument, with a mean cut‐off of ≥10 often used to determine sufficient instrument strength.^(^ ^27^ ^)^ Methods are also available that are robust to the inclusion of weaker instruments in a summary‐level setting. These methods include MR‐Robust Adjusted Profile Score (MR‐RAPS)^(^ ^29^ ^)^ and Genomewide MR Analysis under Pervasive Pleiotropy (GRAPPLE).^(^ ^30^ ^)^ IV2 can be tested for all known and measured confounders in the individual‐level data setting but cannot be tested for unknown or unmeasured confounders. Ensuring summary statistics for summary‐level approaches are drawn from populations of the same genetic ancestry and analyses are statistically adjusted for ancestry will reduce the risk of population stratification.

Several methods have been developed to assess if IV3 is likely to be satisfied. If all instruments are valid, they should all generate the same estimate of the causal effect. In the individual‐level data setting, the Sargan test is used as a measure of heterogeneity in the effect estimates across SNPs,^(^ ^31^ ^)^ whereas Cochran's *Q*‐statistic assesses heterogeneity in an IVW analysis.^(^ ^32^ ^)^ Heterogeneity across instruments can occur due to sampling error, but excessive heterogeneity across instruments suggests that at least some of the instruments are acting on the outcome via a separate pathway. However, it is important to note that a lack of heterogeneity does not necessarily mean all instruments are valid; heterogeneity will also be low if all instruments are invalid in a similar way.^(^ ^9^ ^)^ Another consideration is that heterogeneity can be high for complex traits despite all instruments being valid, as different SNPs instrument different aspects of the trait^(^ ^9^ ^)^ (eg, pain versus structural deterioration in OA).

Alternative methods of meta‐analysis are often additionally performed in the summary‐level setting to assess pleiotropy. Methods that are commonly presented in MR studies are the weighted median and weighted mode estimator. Although the IVW estimate is a weighted mean of all the Wald ratio estimators and is therefore influenced by all Wald ratio estimators, the weighted median estimator is the median Wald ratio estimate and therefore is valid as long as at least 50% of the weight comes from SNPs that are valid (non‐pleiotropic) instruments.^(^ ^33^ ^)^ Similarly, the weighted mode allows invalid instruments, assuming that the invalid instruments will give different effect estimates, whereas the valid instruments will give the same effect estimate. It therefore provides a valid effect estimate as long as the largest group of SNPs are valid instruments.^(^ ^34^ ^)^ The weighted median and weighted mode estimates are therefore often provided alongside the IVW estimate in MR studies, with consistency in effect estimates strengthening the confidence in the IVW estimate. Fig. 3 shows how these methods are commonly presented in MR studies and additional tests that can be performed to test the IV3 assumption.

Perhaps the most commonly used sensitivity method to detect pleiotropy is MR‐Egger regression. Whereas the IVW method sets the intercept of the regression line at zero, the MR‐Egger intercept is allowed to vary from zero.^(^ ^20^, ^35^ ^)^ The intercept represents the average SNP effect on the outcome when the SNP effect on the exposure is zero, thus giving an estimate of directional horizontal pleiotropy.^(^ ^36^ ^)^ The slope of the MR‐Egger regression line can then be considered as a pleiotropy‐robust estimate of the causal effect, although this should not be relied upon as the only effect estimate in an analysis as power, and therefore precision, is lower than the IVW method.^(^ ^35^ ^)^ Although the MR‐Egger assumption relaxes the assumption of no horizontal pleiotropy, it does make an additional assumption that there is no correlation between the SNP effect on the exposure and the pleiotropic effect of the SNP on the outcome, which is often referred to as the Instrument Strength Independent of Direct Effect (InSIDE) assumption.^(^ ^35^ ^)^ In the Section Methods for dealing with correlated pleiotropy, we discuss how this assumption may not hold in cases of correlated exposures and outcomes sharing underlying developmental pathways, for example, when determining the effect of BMD on OA.

Methods that identify outliers can also be used to generate a pleiotropy‐robust estimate of effect.^(^ ^37^ ^)^ For example, MR Pleiotropy Residual Sum and Outlier (PRESSO) identifies pleiotropic SNPs by detecting SNPs that have a greater contribution to the residual sum of squares from the IVW regression.^(^ ^38^ ^)^ These SNPs are then removed when calculating the IVW estimate. However, in some cases, the outlier SNPs may be the best instruments; for example, for molecular traits such as protein expression, the ***cis***‐variant in the gene encoding the protein of interest may actually be the outlier when there are several *trans* instruments,^(^ ^37^ ^)^ however, the ***trans*** instruments are more likely to affect the outcome via distinct pathways. There are several other methods to identify and either remove or downweight outliers in MR analyses, and these methods are reviewed elsewhere.^(^ ^9^, ^37^ ^)^

### Bidirectional MR

MR studies often present bidirectional analyses (ie, reversing the exposure and outcome in a second analysis) for one of two reasons. The first reason being that there is biological plausibility for a causal effect in both directions. For example, an MR analysis determining effects of circulating sclerostin levels on BMD observed an inverse effect, whereby higher plasma sclerostin levels reduced BMD, which is biologically plausible given the role of sclerostin in Wnt signaling inhibition.^(^ ^39^ ^)^ However, the authors also found evidence for an effect of higher BMD on higher plasma sclerostin, suggestive of a feedback mechanism whereby individuals with more bone, and thus more osteocytes, produce more sclerostin to inhibit further bone formation.^(^ ^39^ ^)^ Alternatively, evidence of bidirectional effects may represent horizontal pleiotropy, whereby the instruments are acting through a causal pathway mediated by a common cause of exposure and outcome (see the section on Methods for dealing with correlated pleiotropy). As the effect of an instrument on one trait increases, so will its effect on the other, resulting in evidence of an effect in both directions. An example was our recent analysis of the causal effect of BMD on OA and vice versa, where we observed evidence for an effect in both directions, although the direction of effect of OA on BMD did not reflect the direction we hypothesized for a true bidirectional effect (ie, if OA‐related reductions in physical activity lead to lower BMD).^(^ ^40^ ^)^ Bidirectional MR analyses require summary statistics for SNPs associated with both the exposure and outcome at genomewide significance. Steiger filtering is often performed before analysis to exclude SNPs that explain a greater variance in the outcome variable than the exposure variable for that analysis.^(^ ^41^ ^)^

### Software and data sources

Various software packages and databases have been developed to aid MR studies. MR‐Base is an online platform for performing summary‐level MR analyses, with a linked database of published GWAS summary statistics to aid MR.^(^ ^42^, ^43^ ^)^ Unpublished summary statistics for many traits from UK Biobank, FinnGen, and Biobank Japan have been made publicly available by various groups^(^ ^44^, ^45^, ^46^, ^47^, ^48^ ^)^ and are available to download through this database.^(^ ^43^ ^)^ Published summary statistics can also be downloaded from the GWAS Catalog^(^ ^49^ ^)^ and the Musculoskeletal Knowledge Portal.^(^ ^50^ ^)^ R packages such as TwoSampleMR^(^ ^42^ ^)^ and MendelianRandomization^(^ ^51^ ^)^ have been developed for summary‐level analyses and OneSampleMR^(^ ^52^ ^)^ for individual‐level MR. Stata packages ivonesamplemr^(^ ^53^ ^)^ and mrrobust^(^ ^54^ ^)^ are available for individual‐level and summary‐level analyses, respectively. General IV packages for R (Applied Econometrics with R (AER)^(^ ^55^ ^)^) and Stata (eg, ivreg2^(^ ^56^ ^)^) are often useful for MR estimation.

Given the availability of these tools, which help to simplify MR, there is a danger that researchers can perform and publish MR studies without fully understanding the data sources, instruments, or methodology used. Therefore, when analyzing an MR study, it is important to critically appraise the methodology and interpretation published. Guidelines for performing an MR study have been published,^(^ ^57^ ^)^ and the STROBE‐MR consortium has developed a checklist for MR studies.^(^ ^58^, ^59^ ^)^ These are important to follow when writing an MR article to ensure correct interpretation and reproducibility. These checklists can also form a guide for researchers to assess a published study.

## Developments Relevant to the Musculoskeletal Field

In the first section, we covered the basic concepts of MR to aid researchers in reading and understanding MR literature. In this section, we will give a brief overview of recent developments in MR, which may be useful to consider for researchers aiming to design MR studies in the musculoskeletal field. This is not an exhaustive list of MR developments but a review of the most relevant methods.

### Multivariable and two‐step MR

**Fig. 4:**
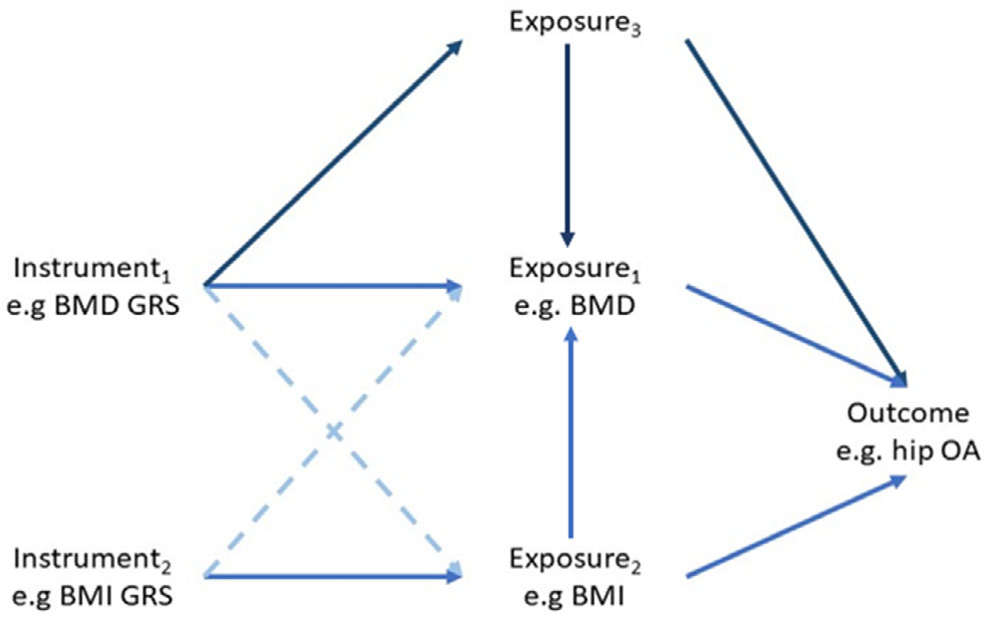
Directed acyclic graph illustrating the concept of multivariable Mendelian randomization. Including additional instruments for body mass index (BMI) in the analysis accounts for the horizontal pleiotropy acting via BMI and gives an estimate of the direct effect of bone mineral density (BMD) on hip osteoarthitis (OA) and BMI on hip OA. However, if there is another pleiotropic pathway via an unknown or unmeasured variable (Exposure_3_), the effect estimates will still be biased. GRS = genetic risk score.

An extension to MR that is becoming more popular in the musculoskeletal field is multivariable MR.^(^ ^1^, ^4^, ^40^, ^60^, ^61^, ^62^, ^63^, ^64^, ^65^, ^66^, ^67^, ^68^, ^69^, ^70^, ^71^, ^72^, ^73^, ^74^, ^75^ ^)^ When evidence of pleiotropy is observed, and there is a trait believed to explain the pleiotropy, multivariable MR can estimate the direct effect of the exposure on the outcome, independent of the additional trait.^(^ ^76^, ^77^ ^)^ For example, in our recent analysis, we used multivariable MR to identify a BMI‐independent effect of BMD on OA, as we suspected that the pleiotropy we observed in our univariable analyses reflected pleiotropy via the shared causal factor, BMI.^(^ ^40^ ^)^ In an individual‐level data setting, multivariable MR requires additional instrument(s) for the second exposure and measurement of the second exposure (ie, the confounding variable) in the same population. In a summary‐level setting, the SNP effects on both exposures and the outcome are required for all instruments. It is not necessary for all instruments to be associated with all exposures, but they should be associated with at least one exposure. However, it should be noted that multivariable MR can only be used to account for correlated pleiotropy if all shared causal factors are included in the model. For example, the analysis in Fig. 4 would still give a biased effect estimate as there is a pleiotropic pathway via Exposure_3_ that has not been accounted for. Multivariable MR also requires that the instruments can robustly predict each exposure, conditional of the other exposures in the model.^(^ ^9^, ^77^ ^)^ This can be determined using the Sanderson‐Windmeijer conditional *F*‐statistic, using a threshold of 10 for each exposure to determine sufficient instrument strength.^(^ ^77^, ^78^ ^)^

Whereas multivariable MR estimates the direct effect of an exposure on an outcome, independent of another exposure, two‐step MR can be used to identify the effect of an exposure on an outcome mediated by another trait.^(^ ^79^, ^80^ ^)^ Two‐step MR involves performing two MR analyses: the first of the exposure on the mediator, then of the mediator on the outcome.^(^ ^80^ ^)^ The indirect effect of the exposure on the outcome, ie, the effect mediated via the mediator, can then be calculated by the product of the beta for the exposure effect on the mediator and the beta for the mediator effect on the outcome.^(^ ^80^ ^)^ The proportion mediated can then be calculated by dividing the indirect effect by the total effect of the exposure on the outcome, estimated by univariable MR. It is important to ensure that any instruments included for the exposure are not related to the mediator and vice versa to reduce the possibility of horizontal pleiotropy.^(^ ^80^ ^)^ An example of the use of this method in the musculoskeletal field was a recent analysis to determine the mediating effect of BMI and smoking on the protective effect of education on rheumatoid arthritis.^(^ ^81^ ^)^

Multivariable MR can also be employed in lifecourse analyses to disentangle the timing at which the exposure influences a later life outcome.^(^ ^82^, ^83^, ^84^ ^)^ For example, a recent multivariable MR analysis identified a direct influence of childhood body size on later life fracture risk, with a higher body size in childhood being protective against fractures, independent of later life body size.^(^ ^83^ ^)^ Conversely, a greater body size in later life appeared to increase fracture risk.^(^ ^83^ ^)^ Two‐step MR was then employed to identify mediators of the effect of childhood body size on later life fracture risk, providing evidence to suggest that adulthood BMD (estimated from heel ultrasound) mediated the effect of childhood body size on later life fracture risk.^(^ ^83^ ^)^

### Novel methods for overlapping samples

Although there is now evidence to suggest that the bias due to sample overlap is minimal in most scenarios,^(^ ^22^ ^)^ the bias toward the observational estimate is greater when instruments are weak,^(^ ^21^ ^)^ for example, using instruments identified in a GWAS where winner's curse is likely to be present (ie, an unreplicated GWAS).^(^ ^22^ ^)^ This is an issue given that many of the publicly available GWAS, available through the data sources described in Software and Data Sources, were performed in UK Biobank. For example, the largest GWAS sample for BMD to date was drawn fully from the UK Biobank population,^(^ ^85^ ^)^ and the population of the largest BMI GWAS to date is also predominantly UK Biobank.^(^ ^86^ ^)^ Therefore, there is the possibility of (i) large sample overlap between exposure and outcome populations and (ii) winner's curse as these GWAS have not been replicated. One could find a previous GWAS with a smaller sample size for the instruments, but this could lead to fewer instruments and lower power. Another consideration for BMD is that previous DXA‐assessed BMD GWAS adjusted for weight,^(^ ^87^, ^88^ ^)^ which can lead to collider bias.^(^ ^89^ ^)^

An online calculator is available to estimate the likely bias and type 1 error rate due to sample overlap,^(^ ^21^ ^)^ and these estimates should be presented in a study presenting two‐sample MR results with overlapping samples. A block‐jackknife approach has recently been described to overcome the limitation of sample overlap and maximize the use of populations such as UK Biobank in an individual‐level data setting.^(^ ^24^ ^)^ This technique involves randomly dividing the total population into a specified number of groups, then excluding each group in turn as a GWAS of the exposure is performed. The resulting independent genomewide significant loci are then used to generate a PRS for the group that was excluded from that GWAS. That way, the instrument for MR was identified in a nonoverlapping population and therefore any weak instrument bias will be toward the null.^(^ ^21^, ^22^ ^)^

### Methods for dealing with correlated pleiotropy

**Fig. 5:**
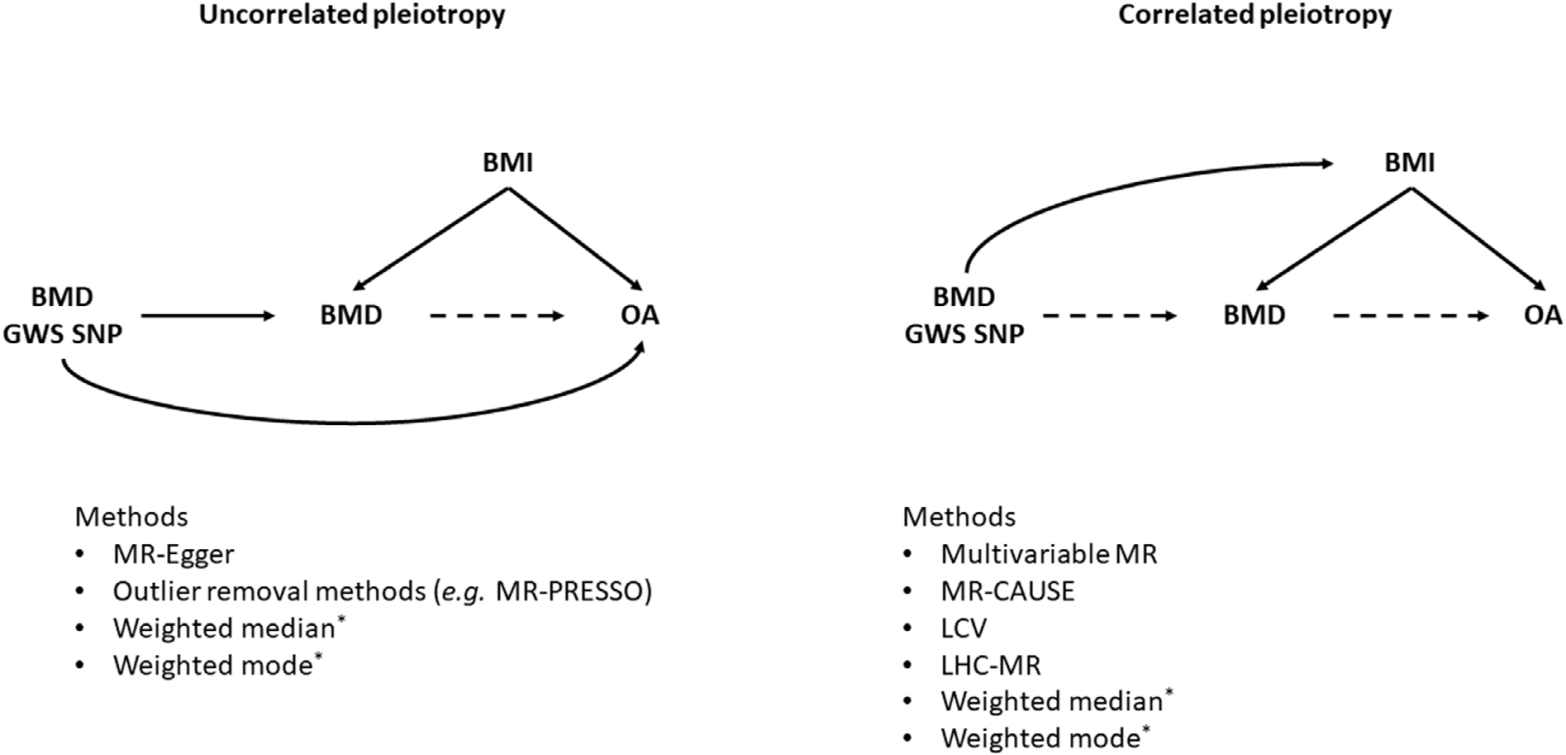
Directed acyclic graphs highlighting the difference between uncorrelated and correlated pleiotropy. *Weighted median and weighted mode can give an unbiased estimate in the presence of either form of pleiotropy, as long as at least 50% SNPs are non‐pleiotropic for the weighted median and the most common Wald ratio comes from the non‐pleiotropic SNPs for the weighted mode. Dashed arrows represent pathways that do not need to be present. BMI = body mass index; BMD = bone mineral density; GWS = genomewide significant; OA = osteoarthritis; SNP = single‐nucleotide polymorphism; MR = Mendelian randomization; LCV = latent causal variable; LHC = latent heritable confounder.

Most of the methods so far described to deal with pleiotropy (except the weighted median and mode methods) are useful to estimating pleiotropy‐robust effect estimates in the presence of **uncorrelated pleiotropy**, where the instrument affects the outcome via a separate pathway to the exposure^(^ ^90^ ^)^ (Fig. 5, left). However, when the instrument affects both the exposure and outcome via a shared pathway, or via a shared causal factor (ie, confounder), **correlated pleiotropy** is present,^(^ ^90^ ^)^ which violates the InSIDE assumption of MR‐Egger.^(^ ^35^ ^)^ If this shared causal factor is known and has been measured, multivariable MR can be performed to estimate the causal effect independent of this confounder.^(^ ^77^, ^90^ ^)^ For example, in the example presented in Fig. 5, a multivariable MR analysis could be performed including both BMD and BMI as exposures.^(^ ^40^ ^)^ However, it is often not possible to identify all common factors contributing to the correlated pleiotropy, and therefore additional MR methods are required.

Methods that have been specifically designed to deal with correlated pleiotropy in MR include the latent causal variable (LCV) method,^(^ ^91^ ^)^ which calculates a genetic causality proportion for one trait on the other without an estimate of the effect; the latent heritable confounder MR model (LHC‐MR), which estimates and quantifies bidirectional effects;^(^ ^92^ ^)^ MR Causal Analysis Using Summary Effect Estimates (CAUSE), which models both correlated and uncorrelated pleiotropy;^(^ ^90^ ^)^ and Welch‐weighted Egger regression, which downweights potential outliers (ie, pleiotropic SNPs) in an Egger regression.^(^ ^93^ ^)^ These methods are more computationally intensive than standard MR analyses as they rely on full GWAS summary statistics for the exposure and outcome (ie, they include all SNPs in the modeling, rather than just genomewide significant instruments). A full review of all novel methods for accounting for correlated pleiotropy is beyond the scope of this review, but we point the readers to the following studies describing additional methods.^(^ ^94^, ^95^, ^96^ ^)^

### MR for disease prognosis/progression

**Fig. 6:**
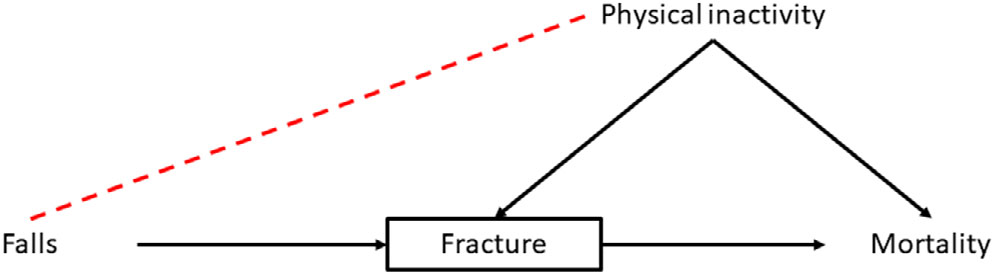
Illustration of the potential issue of collider bias in a study of disease progression/prognosis. The black box around fracture indicates that this variable has been conditioned on by restricting the prognosis (fracture‐related mortality) analysis to individuals who have experienced a fracture. In this hypothetical example, by conditioning on having a fracture, an inverse association between experiencing a fall and level of physical activity may be induced, with those experiencing a fall measuring a higher level of physical activity than fracture cases who have not experienced a fall. If physical inactivity also causes fracture‐related mortality, experiencing a fall may appear protective against mortality. This will also be the case for any instrument associated with experiencing falls. The magnitude and direction of bias will depend on the interaction between the causes of fracture on probability of experiencing a fracture.^(^ ^100^ ^)^

The majority of MR studies in the musculoskeletal field have, to date, focused on causal risk factors for incident disease. However, it is plausible that, for most conditions, there will be different causal risk factors for disease onset and progression^(^ ^97^ ^)^ (eg, progression to total joint replacement in OA). An important consideration in MR of disease progression is collider bias, as the analysis will be restricted to individuals who have incident disease (the full analysis for individual‐level data and the outcome GWAS for summary‐level data). As disease incidence is a common outcome of the disease risk factors, spurious associations between risk factors for disease incidence can be induced in the case population, which can bias the effect estimate for subsequent progression, leading to incorrect causal inference.^(^ ^97^ ^)^ For example, in a study of outcomes after fracture, risk factors for fragility fracture such as physical inactivity or experiencing a fall may be inversely related in the fracture cases, and if one of these risk factors is also causal for post‐fracture mortality, the other risk factor may appear to have an effect on mortality of biased direction or magnitude (Fig. 6).

In an individual‐level setting, inverse probability‐weighted (IPW) two‐stage least‐squares regression can be performed to estimate the effect of an exposure on disease progression accounting for collider bias.^(^ ^97^, ^98^ ^)^ IPW involves modeling the probability of disease incidence using all known predictors for incidence and then weighting the MR analysis by the inverse of this probability.^(^ ^15^ ^)^ In a summary‐level data setting, methods are available to correct the disease progression summary statistics for collider bias, using summary statistics from a GWAS of disease incidence and regression‐based techniques.^(^ ^99^, ^100^, ^101^ ^)^ These corrected summary statistics can then be used to perform collider bias‐free summary‐level MR using methods described above. This relies on the assumption that the instrument‐exposure effect is the same in the diseased compared with the total population, which may not be correct; for example, a recent analysis found that the association between C‐reactive protein (CRP) SNPs and measured CRP differed between cases and controls for obesity and type 2 diabetes.^(^ ^102^ ^)^ A full review of methods to identify and correct for collider bias in MR of disease progression is available elsewhere.^(^ ^103^ ^)^

## Summary and Conclusions

We hope this review has been a useful guide to reading and understanding MR studies or even when designing your own MR study. The MR dictionary, available at https://mr-dictionary.mrcieu.ac.uk/, is a useful further guide to MR concepts. The methodology to perform and test assumptions of MR is constantly evolving, and we recommend the reader strongly considers how the assumptions may or may not be valid with regard to their specific research question. MR is just one method for inferring causality in epidemiological studies, and we hope this review highlights that it comes with its own set of assumptions, which cannot be proven. We therefore highly recommend MR is used as part of a toolkit alongside other methods with different assumptions and biases. This concept is known as triangulation, and we refer the reader to detailed reviews of possible methods for this.^(^ ^104^, ^105^ ^)^

## Conflict of Interest

The authors have no conflicts of interest to declare.

## Disclosures

All authors state that they have no conflicts of interest.

## Author Contributions

**April E Hartley** Conceptualization; writing – review and editing; writing – original draft. **Grace M Power:** Writing – review and editing. **Eleanor Sanderson:** Writing – review and editing. **George Davey Smith:** Conceptualization; writing – review and editing.

### Peer Review

The peer review history for this article is available at https://publons.com/publon/10.1002/jbm4.10675.

## Data Availability

The results presented in Fig. 3 were generated from publicly available data sets accessed through the IEU OpenGWAS project (https://gwas.mrcieu.ac.uk/, data set IDs ieu‐a‐2 and ebi‐a‐GCST007090). No new data were created for this article.
